# P-220. A Needs Assessment of HIV Clinicians in the Evaluation and Initial Management of Kidney Dysfunction Among People Living with HIV: Results from a Cross-Sectional Survey

**DOI:** 10.1093/ofid/ofaf695.442

**Published:** 2026-01-11

**Authors:** Rachel Shulman, Amanda Binkley, Amanda Leonberg-Yoo, Bruce Packett, Adrianne Wyatt, William R Short

**Affiliations:** University of Pennsylvania, Philadelphia, Pennsylvania; Penn Presbyterian Medical Center, North Wales, PA; University of Pennsylvania Hospital System, Philadelphia, Pennsylvania; American Academy of HIV Medicine, Washington, District of Columbia; American Academy of HIV Medicine, Washington, District of Columbia; University of Pennsylvania, Philadelphia, Pennsylvania

## Abstract

**Background:**

Kidney disease is common among people living with HIV (PLHIV). Unique factors associated with HIV, such as sarcopenia and the use of certain antiretroviral therapies, can compromise the accuracy of measurements of kidney function. As primary healthcare providers for many PLHIV, HIV clinicians play a critical role in recognizing and managing kidney disease.

Survey Questions

**Figure:**
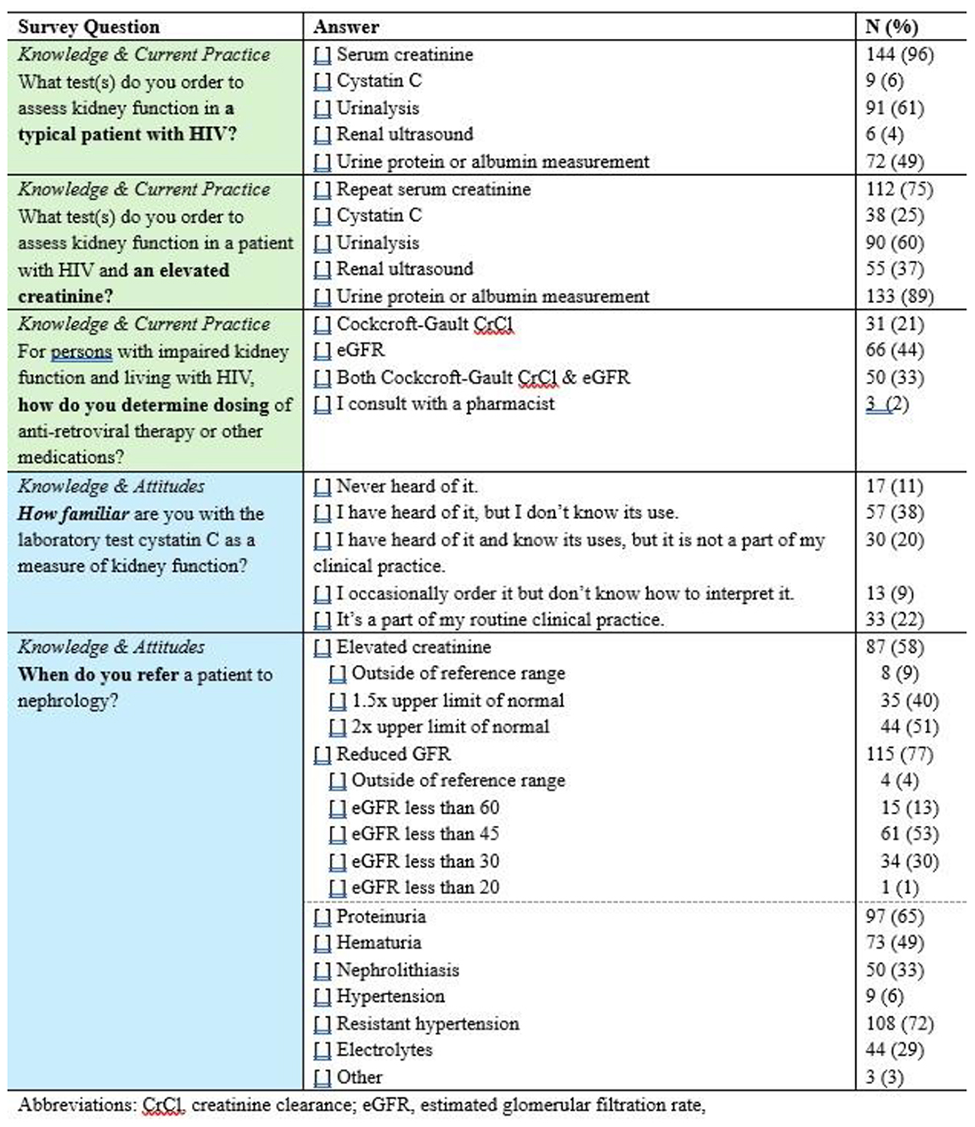

**Methods:**

We distributed an electronic survey to HIV providers via members and/or credentialees of the American Academy of HIV Medicine through a weekly newsletter and two standalone emails sent out biweekly from April 3, 2025 to April 28, 2025. Questions focused on (1) current clinical practices for kidney function assessment, (2) knowledge and attitudes for best clinical practices for kidney function assessment, and (3) identifying care gaps in the management of PLHIV.

**Results:**

A total of 150 providers responded to the survey, including 58 (39%) physicians, 27 (18%) infectious disease pharmacists, 42 (28%) nurse practitioners, and 16 (11%) physician assistants. For routine care of PLHIV, most providers assess kidney function with serum creatinine and urinalysis (96% and 61%, respectively). For PLHIV with an elevated creatinine, 75% of providers (n=112) repeat a serum creatinine, 69% (n=103) measure urine albumin or protein, and 60% (n=90) obtain a urinalysis. Only 25% (n=38) obtain a cystatin C. For drug dosing decisions, 21% of providers (n=31) use Cockcroft Gault creatinine clearance equation, 44% (n=66) use an estimated GFR (eGFR) equation, and 33% (n=50) use both. Among those using an eGFR equation, 37% (n=43) were unsure whether to use the MDRD or CKD-EPI. Only 22% (n=33) of providers felt comfortable incorporating cystatin C into eGFR assessment. There was variable practice in nephrology referral. Of the 115 of providers who refer to nephrology for reduced eGFR, 13% (n=15) referring at an eGFR< 60mL/min/1.73m^2^ and 53% (n=61) at 45mL/min/1.73m^2^. Only 30% (n=34) waited until 30mL/min/1.73m^2^.

**Conclusion:**

In this survey, HIV providers reported high rates of uncertainty regarding evaluation of elevated creatinine and GFR assessment. Targeted educational intervention to reduce medication errors related to drug dosing as well as appropriate referrals to nephrology

**Disclosures:**

Amanda Binkley, PharmD, BCIDP, AAHIVP, Shionogi: Advisor/Consultant William R. Short, MD, Gilead Sciences: Grant/Research Support|ViiV: Advisor/Consultant

